# Comparison of translabial three-dimensional ultrasonography and magnetic resonance imaging for the grading of levator ani defects

**DOI:** 10.1097/MD.0000000000025997

**Published:** 2021-05-21

**Authors:** Yijia Luo, Linxin Yang, Ning Lin, Zhihua Fan

**Affiliations:** a Shengli Clinical Medical College of Fujian Medical University; b Department of Ultrasound, Fujian Provincial Hospital, Fuzhou, China.

**Keywords:** levator ani defect, modeling reconstruction, magnetic resonance imaging, pelvic floor, three-dimensional ultrasound

## Abstract

Levator ani defect (LAD) closely correlates with pelvic organ prolapse. This study aimed to compare the LAD grading between 3-dimensional ultrasonography (3D-US) and magnetic resonance imaging (MRI) and investigate the reasons for the difference using 3-dimensional pelvic models.

Seventy-two Chinese women who were to undergo repair surgery were assessed by the prolapse staging, 3D-US and MRI. LAD was graded according to the grading systems described with regard to 3D-US (Dietz et al.) and MRI (Delancey et al.) The puborectalis attachment width and the puborectalis thickness were measured on the reconstructed pelvic models offline within the software. The results were analyzed using the weighted kappa and the ANOVA test.

The grading systems used for 3D-US and MRI showed the good agreement (κ = 0.75), whereas the consensus of the extent (ie, partial or complete) of tears showed the moderate agreement (κ = 0.56). Additionally, iliococcygeus tears detected by MRI (n = 3) accompanied with complete puborectalis tears on the same side. The averaged width of intact puborectalis attachment was 13.75 ± 3.43 mm. The width of intact puborectalis attachment was remarkably higher than that of the injured attachment (*P* = .005). The averaged puborectalis thickness was 9.85 ± 2.13 mm.

Comparison of 3D-US and MRI showed the good agreement on LAD grading. The moderate agreement in assessing partial or complete tears resulted from the grading criteria of 3D-US. The morphological characteristics of puborectalis assisted in identifying complete tears.

## 1 Introduction

Levator ani muscle (LAM) plays a key role in supporting pelvic organs and maintaining pelvic functions, which has three major components, puborectalis, iliococcygeus and pubovisceral muscle.^[1]^ It was established that the levator ani defect (LAD) increased the risk of pelvic organ prolapse (POP), especially significant anterior and central compartment prolapse.^[2,3]^ Additionally, the size of the defect has a direct correlation with symptoms of prolapse.^[4]^ It is significant to grade LAD accurately for the better treatment and the investigation of POP mechanism.

LAD can be detected by digital palpation, magnetic resonance imaging (MRI) and three-dimensional ultrasonography (3D-US). Digital palpation, as a subjective method, requires substantial teaching and appears limited reproducibility.^[5]^ Although there is no gold standard is available, MRI is considered as the most reliable reference because of its intense soft tissue contrast and discriminatory competence.^[6,7]^ However, two-dimensional magnetic resonance images cannot provide sufficient information about LAM. Within the specific postprocessing software, 3-dimensional magnetic resonance-based models (3D-MR-model) can provide the stereoscopic overview and valid details of the complex anatomy. Meanwhile, 3D-US has emerged as an easily accessible and cost-effective alternative to MRI.^[8,9]^ The most widely used grading systems for LAD were described with regard to MRI (Delancey et al) and 3D-US (Dietz et al).^[2,8,10]^ Both grading systems showed good interrater reliability.^[11,12]^

So far, few studies have compared the 3D-US and MRI on the grading of LAD. In addition, the reasons for the difference between two imaging methods on LAD grading have remained unclear. Therefore, aimed at women with POP, this study compared the 3D-US and MRI on LAD grading. The second objective was to investigate the reasons for the difference with the use of 3D-MR-models.

## 2 Materials and methods

This research was conducted in Fujian Provincial Hospital from October 2019 and February 2020. It was approved by the Ethics Committee affiliated with Fujian Provincial Hospital. All participants signed the informed consent.

A total of 72 Chinese women who were to undergo prolapse repair surgery were included from the urogynecology clinic. Before the imaging examinations, women were interviewed about symptoms of urinary incontinence, prolapse, or fecal incontinence using a standardized questionnaire.^[13]^ They also underwent the prolapse staging with the use of the grading system of the International Continence Society.^[14]^ The exclusion criteria were:

(1) history of abdominal or pelvic surgery;
(2) history of pelvic inflammatory adhesion;
(3) history of metal implantations;
(4) claustrophobia;
(5) inability to understand the instruction given in Mandarin.

The time interval between the examinations of 3D-US and MRI was 1 to 5 days.

### 2.1 Ultrasonography examinations

**Figure 1:**
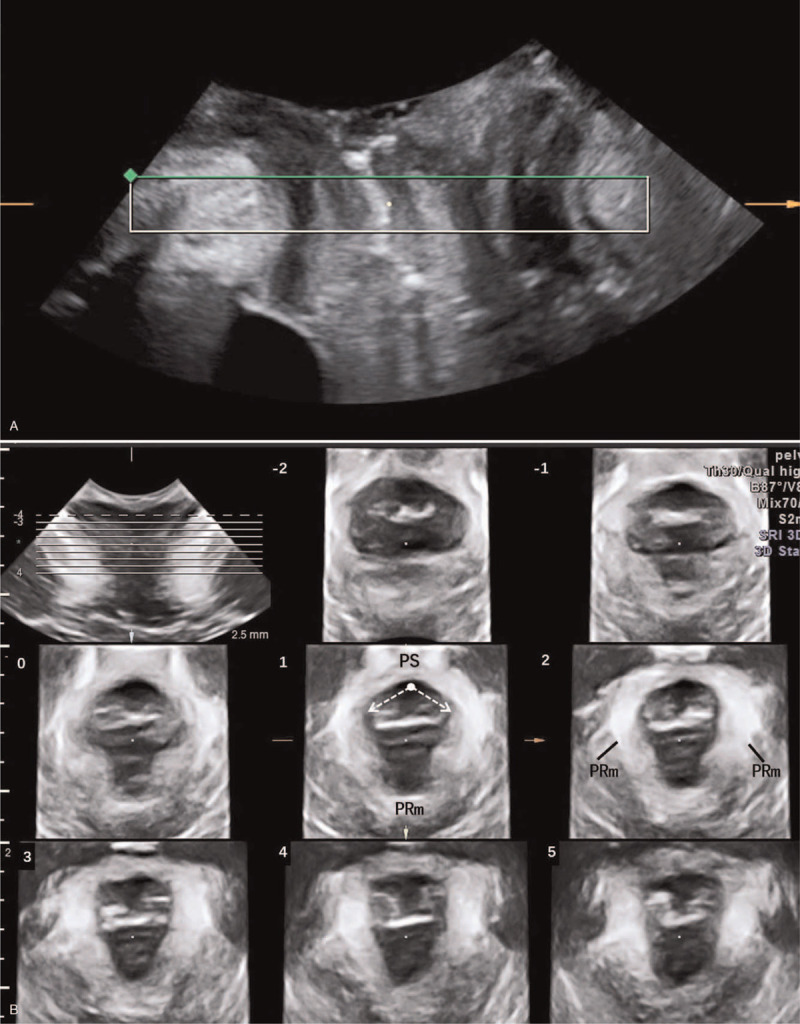
(A) In midsagittal plane, the plane of the minimal hiatal dimension is identified between the hypoechoic posterior margin of pubic symphysis and hyperechoic anterior border of puborectalis. (B) The perineal view of the levator hiatus obtained by tomographic ultrasound imaging (TUI), from slice -2 to slice 5. White arrows show the measurement of levator-urethra gap (LUG). PS, pubic symphysis; PRm, puborectalis.

Translabial ultrasonography was performed by a GE Voluson E8 system (GE Kretz technik GmbH, Zipf, Austria) with a RAB 4–8 MHz volume transducer. The patients were supine in the lithotomy position during the examinations. In the midsagittal plane, the minimal hiatal dimension was identified between the hypoechoic posterior margin of pubic symphysis and hyperechoic anterior border of puborectalis (Fig. 1A). The volume datasets for tomographic ultrasound imaging (TUI) were acquired above the minimal hiatal dimension during the pelvic floor maximum contraction (PFMC) (Fig. 1B). The patients who were unable to complete the PFMC were excluded. The obtained images were analyzed by 2 senior doctors offline, who were blinded to each other. If there were conflicts in diagnosis, they discussed to make consensus.

### 2.2 MRI examination

The MRI examinations were performed on the patients in the supine positions. The magnetic resonance images were acquired by a high-resolution axial 3T scanner (Siemens, Erlangen, Germany) equipped with a 35-cm field of view. Standard imaging for pelvic floor was performed with the axial T2-weighted turbo spin echo sequence (TR, 1260 ms; TE, 130 ms; slice thickness, 1.0 mm).^[9]^ The acquired MRI datasets were evaluated offline by 2 experienced radiologists separately, who were blinded to clinical information and made consensus through discussion.

### 2.3 Three-dimensional modeling

**Figure 2:**
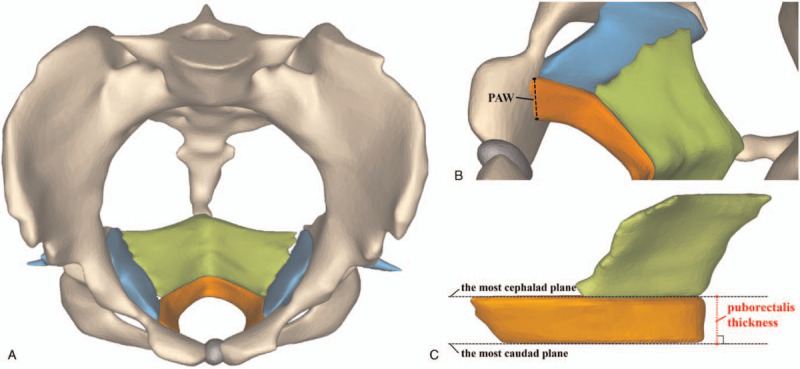
(A) The three-dimensional model of pelvic floor. (B) The measurement of the puborectalis attachment width (PAW). (C) The measurement of the puborectalis thickness. Showing muscles and pelvic bone in color, the puborectalis in orange, the iliococcygeus in green, the internal obturator muscle in blue, the pelvic bone in light yellow and the pubic symphysis in grey.

MRI datasets of the patients were imported into Mimics 17.0 (Materialise Group, Leuven, Belgium). According to anatomical descriptions,^[1]^ the puborectalis, iliococcygeus, internal obturator muscle, pubic ramus and pubic symphysis were delineated on successive axial MRI scans, which could be displayed in 3D rendering (Fig. 2A). A range of advanced imaging processing methods were applied to 3D objects, such as smoothing and wrapping. The puborectalis attachment width (PAW) was measured as the depth of origins of each levator sling from the pubis inner surface, which was taken from inner surface of puborectalis (Fig. 2B).^[15]^ The puborectalis thickness was measured as the vertical distance between the most cephalad plane and the most caudad plane of the puborectalis, which was taken at the distal posteromedial portion of puborectalis (Fig. 2C). According to the anatomy, the most caudad plane on puborectalis model (Fig. 2C) was equal to the axial plane of the minimal hiatal dimension on 3D-US (Fig. 1A). The measurement was performed 3 times, and mean values were recorded for statistical analysis.

### 2.4 Comparisons of two grading systems

During the PFMC, a set of eight parallel slices was acquired in the axial plane at 2.5-mm intervals by 3D-US, which was from 5 mm below to 12.5 mm above the plane of minimal levator hiatus dimension (Fig. 1B). According to 3D-US grading system (Dietz et al), the complete tear was defined if all three central slices (slice 0 to 2) presented abnormal insertions, while the partial tear was defined if any of slice the third to eighth slices (slice 0 to 5) showed abnormal except what was diagnosed with complete tear (Fig. 1B).^[8]^ In equivocal cases, the levator urethra gap (LUG) was used. Measurements of the LUG were carried out by placing calipers in the center of the hypoechogenic structure that indicates the urethral mucosa and smooth muscle and on the most medial aspect of the muscle insertion.^[8]^ The insertion was regarded as the abnormity when the LUG was greater than 25 mm.^[8]^

According to MRI grading system (Delancey et al.), LAD was defined as a discontinuity between the puborectalis and the inferior pubic ramus (at least one 4-mm section or two and more adjacent 2-mm sections in both the axial and coronal planes).^[10]^ LAD was classified into intact (no visible damage), low-grade tear (fiber loss < 50%), high-grade tear (fiber loss of > 50%) and complete tear (no residual fiber remained).^[2,10]^

Direct comparison of MRI and 3D-US for LAD is limited due to the different grading systems. Therefore, two grading systems were adjusted to 3-point scale grading systems. Both grading systems assessed each side of the puborectalis separately. LAD was divided into intact, partial tears and complete tears.

### 2.5 Statistical analysis

The statistical analysis was performed by SPSS 17.0 for Windows (SPSS Chicago, IL). The agreement between two grading systems and the interobserver agreement on 3D-US and MRI were assessed by Cohen's kappa. The value of κ less than 0.20 indicates poor, 0.21–0.40 fair, 0.41–0.60 moderate, 0.61–0.80 good, and 0.81–1.00 excellent agreement. The parameters were presented as the mean positive and negative standard deviation ($\bar{\text{X}}$± s). These parameters between groups were compared by the ANOVA test. A value of *P* < .05 was considered statistically significant.

## 3 Results

Because of the exclusion criteria, eleven patients were excluded. Then 61 patients to be assessed. The general demographic characteristics of patients were shown in Table 1. The mean age was 62.1 years. Fifty-eight patients had undergone vaginal deliveries. Complaints at presentation were prolapse symptoms (90%), urinary stress incontinence (33%) and symptoms of voiding dysfunctions (26%).

**Table 1:** General demographics of women with pelvic organ prolapse (N = 61).

Variables	Mean (range)
Age (yr)	62.1 (40–87)
Body mass index (kg/m^2^)	23.2 (19.6–33.2)
Parity (n)	3.1 (0–8)
Pelvic organ prolapse grade	Number (Number/total %)
I	0
II	9 (14.8%)
III	29 (47.5%)
IV	23 (37.7%)

On MRI, 14 patients were diagnosed with unilateral LAD (6 on the left, 8 on the right), while 10 had bilateral LAD. According to the MRI grading system, 22 attachments presented partial tears (12 with low-grade tears, 10 with high-grade tears). Meanwhile, 12 attachments showed complete tears. It was found that the iliococcygeus was detached from the arcus tendinous of levator ani (n = 3) accompanying with the complete puborectalis tears on the same side. The interobserver agreement between two radiologists in LAD grading on MRI had a κ of 0.69 (95% Confidence interval [CI] 0.55–0.84), which was defined as good agreement.

On 3D models, the PAW of intact attachments (n = 87) reached 13.75 ± 3.43 mm, ranging from 6.15 mm to 22.04 mm. The PAW of low grade tears (n = 12) was 10.77 ± 3.07 mm, while the PAW of high-grade tears (n = 10) was 4.66 ± 0.97 mm. The PAW of three groups (ie, intact, high-grade tears and low-grade tears) had significant statistical differences (*P* = .005 < .05). The puborectalis thickness was 9.85 ± 2.13 mm, ranging from 3.22 mm to 18.33 mm.

On 3D-US, 9 patients had bilateral LAD and 14 patients had unilateral LAD (7 on the left, 7 on the right). Based on the 3D-US grading system, 12 attachments showed partial tears, while 21 attachments presented complete tears. The κ statistic for showing interobserver agreement for two observers in LAD grading on 3D-US was 0.77 (95% CI 0.62–0.91), as good agreement.

In the Table 2, the grading of MRI and ultrasound on LAD were compared. Comparison showed good agreement, with a weighted kappa of 0.75 (95% CI, 0.64–0.87). The diagnosis of defect or not showed the excellent agreement, with a weighted kappa of 0.888 (95% CI, 0.76–0.96). Whereas the diagnosis of partial or complete tears showed the moderate agreement, with a weighed kappa of 0.56 (95% CI, 0.31–0.80).

**Table 2:** Comparison of the grading of 3-dimensional ultrasound and magnetic resonance imaging on levator ani defects.

		Ultrasound grading (Dietz et al)			
		Intact	Partial tear	Complete tear	Total
MRI grading (Delancey et al)	Intact	86	1	1	88
	Partial tear	4	11	7	22
	Complete tear	0	0	12	12
	Total	90	12	20	122

**Figure 3:**
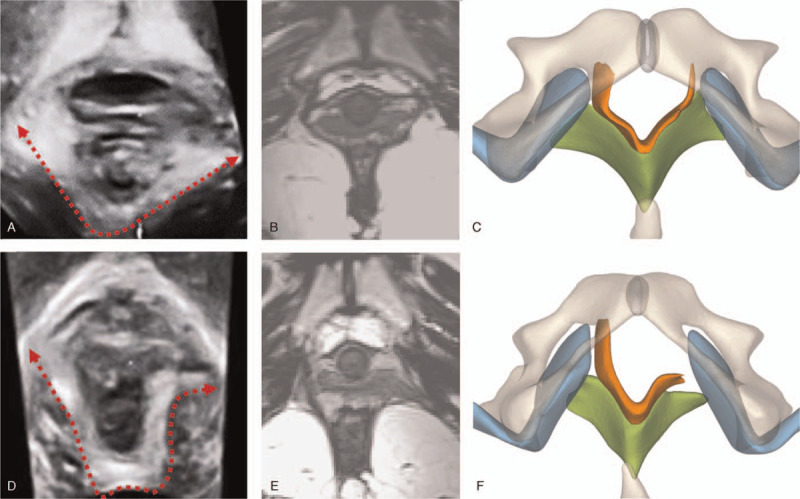
The ultrasound images, magnetic resonance images and three-dimensional models of the two cases are shown (A-F). Each line represents the same case respectively. Images in the first column are ultrasound images, images in the second column are magnetic resonance images, and images in the third column are three-dimensional pelvic models. Images in the first line (A-C) show the left puborectalis tear (a complete tear on 3D-US, a partial tear on MRI). The images in the second line (D-F) show the left puborectalis tear with iliococcygeus tears (a complete tear on 3D-US and MRI). Red dotted lines show the morphology of the puborectalis (A, D). Showing muscles and pelvic bone in color, the puborectalis in orange, the iliococcygeus in green, the internal obturator muscle in blue.

Seven of 20 (35%) with complete tears on 3D-US was defined as partial tears on MRI (Fig. 3A, 3B). Four with partial tears on MRI were missed on 3D-US. On the three central images on the TUI, the puborectalis with no defect was continuous and formed a V-shaped sling running from the pubic ramus towards the anorectal junction (Fig. 1B). Meanwhile, the performance of puborectalis with defects could be divided into two types, the injured ends of the ‘V’ keeping straight (Fig. 3A, 3B, and 3C) or distorting laterally (Fig. 3D, 3E, and 3F). According to our analysis, when the LUG>25 mm on all the three central slices of TUI was diagnosed as complete tears, the diagnostic sensitivity reached 100% and the diagnostic specificity was 85%. If the injured end of puborectalis distorted laterally on all the three slices was diagnosed as complete tears, the diagnostic sensitivity was 100% and the diagnostic specificity was 97.3%. If the injured end of puborectalis distorted laterally as well as the LUG>25 mm on all the three slices were diagnosed as complete tears, the diagnostic sensitivity kept 100% and the diagnostic specificity was promoted to 99.1%.

## 4 Discussion

The LAM consists mainly of puborectalis, which spans the pelvic outlet.^[1,3]^ The puborectalis has two ends attached to the pelvis of variable thickness and forms the levator hiatus with pelvic ramus.^[1,15]^ The urethra, vagina and rectum exit the pelvis through the levator hiatus. The intact LAM can prevent the overactivity of pelvic organs and provide the upward support for pelvic organs for minimizing the risk of POP.^[16]^ During the vaginal delivery, the fetus exits the pelvic floor through the levator hiatus and the puborectalis has to stretch substantially.^[17]^ Given the degree of muscle stretch involved, it is not surprising that vaginal delivery may lead to muscle trauma. When the puborectalis detaches from the pelvis, the PAW and the puborectalis thickness decrease, the strength of support is weakened, and the dimension of levator hiatus increase, which contribute to the pelvic organs (bladder, womb and rectum) prolapse.

Previous researches compared 3D-US with MRI on the grading of LAD. In the single center study, Zhuang et al and Vergeldt et al found that two imaging methods showed good agreement but was lowest for the highest-grade defects.^[18,19]^ In the multicenter study of 135 scans, Notten et al also concluded the agreement in recognizing the major levator ani defects was moderate.^[9]^ The results of previous studies corresponded with this study. However, these studies just roughly provided speculations about the reasons for the disagreement on the LAD grading without analysis and verifications. The major speculation was that the disagreement resulted from the scoring criteria rather than the imaging method itself. Although this study is a single center study and the sample size was no larger than prior studies, this is the first study used 3D-MR-models to investigate and analyze the reasons for the difference on LAD grading.

The majority of previous studies used the measurements on the 2-dimensional planes of 3D-US and MRI to describe the morphology of the puborectalis.^[20,21]^ There were few studies reconstructing the 3-dimensional puborectalis and measuring the spatial parameters for anatomical description. This study used techniques to reconstruct the 3D-MR-models of pelvic floor and measured spatial parameters for better describing the LAM morphologies of POP women.

We considered that the moderate agreement of the extent mainly resulted from the 3D-US grading criteria. Delancey et al quantified the amount and the ratio of muscle loss to diagnose complete or partial tears by MRI.^[10]^ Dietz et al observed three central slices on TUI to judge complete or partial tears during PFMC.^[8]^ However, during PFMC, three central slices showed 5-mm vertical distance above the minimal hiatal dimension (Fig. 1), which equaled to the extent of puborectalis at 5-mm thickness above the most caudad plane (Fig. 2C). As we know, the fibers of puborectalis would shorten and the thickness of puborectalis would increase during PFMC. Therefore, during PFMC, the puborectalis thickness and the PAW would be higher than at rest. The puborectalis thickness was 9.85 ± 2.13 mm, ranging from 3.22 mm to 18.33 mm. The puborectalis thickness of 77% patients (47/61) was higher than 5 mm. It was indicated that the three central slices on TUI were not sufficient for assessing complete tears. According to our analysis, if we defined the injured end of puborectalis distorted laterally on all the three central slices of TUI as complete tears, the diagnostic specificity promoted from 85% to 97.3%. Moreover, if we defined the free end of puborectalis distorted laterally as well as the LUG>25 mm on all the three slices as complete tears, the diagnostic specificity promoted from 85% to 99.1%. Theoretically, muscle tears range from a loss of a few muscular fibers (partial tears) to disruption of all the muscle (complete tears). It is expected that the puborectalis with partial tears can keep the V-shape because of the residual fibers attached to the pubic ramus (Fig. 3C), which still support the pelvic organs. Whereas the complete tears destroy the V-shape and is difficult to reverse. The free-ends of the puborectalis shorten towards the dorsal side. Lack of the limit and support of the puborectalis, the pelvic organs, like the urethra, bladder and vagina would bulge laterally or caudally (Fig. 3F). The bulged pelvic organs will squeeze the free-end of puborectalis and result in the lateral distortion. The novel finding in this study would contribute to revising the present grading criteria of 3D-US.

The true gold standard is the vivisection with microscopic correlation, which is impractical at present. It was reported excellent correlation between cadaveric structures and MRI anatomy,^[6]^ so MRI could be the only other optimal assessment tool for LAD. MRI is widely recognized as the most reliable imaging tool for LAD.^[22]^ In this study, three cases showed that the iliococcygeus was detached from tendinous arch of levator ani on MRI. Because the location of iliococcygeus had a certain distance from the body surface, which was beyond the observation of the translabial ultrasonography. Compared with 3D-US, MRI is able to classify the size accurately. However, its price and contraindications inhibit its clinical promotion. Translabial 3D-US is the simple and accessible method, non-invasive, economic and has the caliber of providing a real-time assessment of pelvic floor, even though the suboptimal interobserver agreement seems to limit its clinical application. 3D-US showed the good agreement with MRI in LAD grading, whereas manifested moderate agreement in classifying partial or complete tears.

There were several limitations in this study. Firstly, the parameters from non-prolapse women were not included in this study which could better describe the pelvic floor. Secondly, the number of the attachments with defect showed lower than the intact comparatively. In the future study, we would expand the sample size of the attachments with defect for verification of the conclusions. Thirdly, this sample size was not enough to analyze whether iliococcygeus tears accompanied with puborectalis complete tears on the same side, which would be discussed in the future work.

## 5 Conclusions

3D-US and MRI had the good agreement in the grading of LAD. The extent (ie, partial or complete) of tears showed the moderate agreement. According to the measurements from 3D-MR-models, it was illustrated that the criteria for complete tears on 3D-US resulted in the moderate agreement. The morphological characteristics of puborectalis would assist in discriminating partial or complete tears on 3D-US.

## Author contributions

**Conceptualization:** Yijia Luo.

**Data curation:** Linxin Yang, Zhihua Fan.

**Formal analysis:** Yijia Luo, Ning Lin.

**Funding acquisition:** Ning Lin.

**Methodology:** Linxin Yang.

**Project administration:** Ning Lin.

**Resources:** Ning Lin.

**Software:** Yijia Luo, Linxin Yang.

**Supervision:** Zhihua Fan.

**Writing – original draft:** Yijia Luo.

**Writing – review & editing:** Yijia Luo, Zhihua Fan.
